# Cardioprotective Effects of Taurisolo® in Cardiomyoblast H9c2 Cells under High-Glucose and Trimethylamine N-Oxide Treatment *via De Novo* Sphingolipid Synthesis

**DOI:** 10.1155/2020/2961406

**Published:** 2020-11-12

**Authors:** Stefania Lama, Vincenzo Monda, Maria Rosaria Rizzo, Marco Dacrema, Maria Maisto, Giuseppe Annunziata, Gian Carlo Tenore, Ettore Novellino, Paola Stiuso

**Affiliations:** ^1^Department of Precision Medicine, University of Campania “Luigi Vanvitelli”, Naples, Italy; ^2^Department of Experimental Medicine, Section of Human Physiology, University of Campania “Luigi Vanvitelli”, Naples, Italy; ^3^Department of Advanced Medical and Surgical Sciences, University of Campania “Luigi Vanvitelli”, Naples, Italy; ^4^Department of Pharmacy, University of Naples Federico II, Naples, Italy

## Abstract

In addition to high plasma glucose, increased levels of trimethylamine N-oxide (TMAO) have been found in obese subjects, where are considered as a novel risk factor for cardiovascular diseases. The present study aimed to investigate the effect of a novel nutraceutical formulation based on grape polyphenols (registered as Taurisolo®) in counteracting TMAO- and high glucose (HG)-induced cytotoxicity in cardiomyoblast H9c2 cells. Cell damage was induced with HG (HG-H9c2) and HG+TMAO (THG-H9c2); both experimental cell models were, thus, incubated for 72 h in the presence or absence of Taurisolo®. It was observed that Taurisolo® significantly increased the cell viability and reduced lactate dehydrogenase and aspartate transaminase release in both HG- and THG-H9c2 cells. Additionally, through its antioxidant activity, Taurisolo® modulated cell proliferation *via* ERK activation in THG-H9c2. Furthermore, Taurisolo® was able to induce autophagic process *via* increasing the expression of LC3II, a protein marker involved in formation of autophagosome and *ex novo* synthesis of sphingomyelin, ceramides, and their metabolites both in HG- and THG-H9c2 cells. Finally, Taurisolo® reduced hypertrophy and induced differentiation of HG-H9C2 cells into cardiomyocyte-like cells. These data suggest that Taurisolo® counteracts the toxicity induced by TMAO and HG concentrations increasing autophagic process and activating *de novo* sphingolipid synthesis, resulting in a morphological cell remodeling. In conclusion, our results allow speculating that Taurisolo®, combined with energy restriction, may represent a useful nutraceutical approach for prevention of cardiomyopathy in obese subjects.

## 1. Introduction

Obesity is a medical condition mainly characterized by increased body fat accumulation, which negative effects on health are well-known. Excessive adipose tissue is among the leading causes of chronic inflammation that, in turn, plays a pivotal role in developing or worsening the outcome of various pathologies such as metabolic, cardiovascular, respiratory, viral, and tumoral [1]. Obesity is a widely spread health problem in the world, and it has been recognized as a risk factor for the development of metabolic disorders, such as type 2 diabetes, cardiovascular disease, or atherosclerosis [2, 3]. Diet-induced obesity (DIO) leads a significant increase of oxidative stress (OS) and reduced antioxidant defense, and it is associated with a typical chronic low-grade inflammation, which results in severe cardiovascular complications [4, 5]. Moreover, in cardiomyocytes, DIO induced many alterations, including mitochondria dysfunction [5], endoplasmic reticulum stress [6], apoptosis, and autophagy. Many studies suggest that enhanced autophagy acts as a protective mechanism against OS in the liver, adipose tissue, and skeletal muscle [7, 8]. Furthermore, when cardiac stresses are sustained for a prolonged period, e.g., in high blood sugar levels, upregulation of autophagy promotes myocyte cellular architecture remodeling. Sphingolipids (SLs), a heterogeneous lipid class that includes ceramide (Cer), sphingosine-1-phosphate, and dihydroceramide present in all membrane structures, have been associated to mediate distinct autophagic pathways described as protective autophagy and autophagy-associated cell death [1, 9]. A recent study showed that Cer directly interacts with microtubule-associated protein light chain 3 (LC3) on mitochondrial membranes, inducing autophagy [10]. Therefore, Cer has been shown to reduce the nutrient transporters in plasma membrane resulting in autophagy activation. S1P has emerged as a cell-proliferative lipid messenger. It has been found to induce survival-mediated or protective autophagy under nutrient starvation, but it was shown to not be related to Beclin1 protein accumulation or class I PI3K or Akt suppression [11, 12]. The conversion of Cer to S1P simultaneously accumulates the survival effects and removes the death signals. This observation led to the concept of a so-called “SL rheostat” or “SL biostat,” based on the relative amounts and reciprocal roles of these antagonistic metabolites, which are critical in guiding the destiny of cells [12–14].

Circulating trimethylamine N-oxide (TMAO) levels has been recently highlighted as a potential prognostic marker for cardiovascular diseases (CVD). Increased TMAO concentrations have also been associated with impaired glucose tolerance and diabetes and were accompanied by other metabolic conditions such as low HDL cholesterol and phospholipids and hypomethylation [15]. Recently, Annunziata et al. demonstrated the ability of a novel nutraceutical formulation based on grape pomace polyphenolic extract (registered as Taurisolo®) to reduce the serum levels of TMAO in healthy [16] and in overweight/obese subjects [17]. The authors have hypothesized two mechanisms of action by which Taurisolo® may exert its TMAO-reducing effect: the antioxidant activity and the microbiota remodeling, both exerted by polyphenols. The goal of the present study was to investigate the *in vitro* effect and signaling mechanisms of Taurisolo® on proliferation, OS, morphological remodeling, and autophagy, in trimethylamine TMAO and hyperglycemia damaged embryonic rat cardiomyoblast H9c2 cells.

## 2. Materials and Methods

### 2.1. Chemicals

All chemicals, standards, and reagents used were either analytical or mass grade reagents. The water was treated in a Milli-Q water purification system (Millipore, Bedford, MA, United States) before use. Bovine serum albumin (BSA) and 3-(4,5-dimethylthiazol-2-yl)-2,5-diphenyl tetrazolium bromide (MTT) and trimethylamine N-oxide (TMAO) were purchased from Sigma-Aldrich (St. Louis, MO, USA). Phosphate-buffered saline (PBS) and trypsin-EDTA were from Lonza (Milano, Italy). Fetal bovine serum (FBS) and Dulbecco's modified Eagle's medium (DMEM) were purchased from Gibco (Grand Island, NY, USA). Western blot analysis was performed using the following primary antibodies: polyclonal antibody (polyAb) p-ERK 44/42 and polyAb ERK 44/42 from Cell Signaling Technology (Beverly, MA, USA); monoclonal Ab Mn-SOD was from Santa Cruz Biotechnology (San Diego, CA, USA). Blots were incubated with horseradish peroxidase (HRP)-conjugated goat anti-rabbit or HRP-conjugated goat anti-mouse (Immunoreagents Inc., Raleigh, NC, USA) secondary antibodies. Anti-mAb was purchased from Santa Cruz Biotechnology and secondary antibodies conjugated to Alexafluor 488 from Life Science, Portland, OR, USA, and 4′,6-diamidino-2-phenylindole (DAPI) from Sigma-Aldrich.

### 2.2. Taurisolo® Supplement

Taurisolo® is a supplement consisting of a polyphenol extract obtained from *Aglianico* cultivar grape, collected during the autumn 2016 harvest. Firstly, the Department of Pharmacy, University of Naples Federico II (Naples, Italy), provided the supplement formulation; then, the large-scale production was accomplished by MBMed Company (Turin, Italy). For the polyphenol extract production, grapes were extracted with water (50°C), and the solution was filtrated and concentrated and underwent a spray-drying process with maltodextrins as support (40-70%) to obtain a fine microencapsulated powder. As previously reported, the High-Performance Liquid Chromatography-diodearray detector (HPLC-DAD, Jasco Inc., Easton, MD, USA) polyphenol profile of Taurisolo® revealed the presence of: Ferulic acid 10.5 ± 0.70 *μ*g/g, Resveratrol 13.6 ± 0.64 *μ*g/g, Caffeic acid 20.7 ± 0.76 *μ*g/g, Procyanidin B3 dimer 22.05 ± 6.61 *μ*g/g, p-coumaric acid 27.9 ± 0.66 *μ*g/g, Rutin 28.4 ± 0.70 *μ*g/g, Quercetin 40.22 ± 7.11 *μ*g/g, Procyanidin C2 trimer 44.6 ± 0.66 *μ*g/g, Procyanidin B4 dimer 56.6 ± 0.88 *μ*g/g, Procyanidin B1 dimer 62.8 ± 0.59 *μ*g/g, Procyanidin B2 dimer 426.5 ± 5.92 *μ*g/g, Syringic acid 539.2 ± 6.02 *μ*g/g, Epicatechin 886.0 ± 7.82 *μ*g/g, Gallic acid 1463.4 ± 65.5 *μ*g/g, and Catechin 4087.0 ± 64.5 *μ*g/g [18].

### 2.3. Cell Culture

Rat cardiomyocytes (H9c2) (ATCC, Manassas, VA) cells were cultured in DMEM, at two different glucose concentration 5.5 mM (NH-H9c2) and 44 mM (HG-H9c2), supplemented with 10% fetal bovine serum, 100 U/mL of penicillin, and 100 lg/mL of streptomycin in 150 cm^2^ tissue culture flasks at 37°C in a humidified atmosphere of 5% CO_2_. The cells were fed every 2–3 days and subcultured once they reached 70–80% of confluence. After 4 hr incubation, cells were washed with 1% PBS to remove unattached dead cells and treated with Tau (0,5 *μ*g/*μ*L), TMAO (50 *μ*M), and TMAO/Tau combination.

### 2.4. Cell Proliferation Assay

The evaluation of cell proliferation was performed on NG-H9c2 and HG-H9c2 cell line after 24 and 72 hr incubation with Taurisolo®, TMAO, and TMAO/Tau combination. The cells were seeded in 96-well plates in a number of 30 × 10^2^ per well. The growth was assessed by MTT viability assay as previously described [19]. Then, MTT assay was carried out by triplicate determination on at least three separate experiments. All data were expressed as mean ± SD. We have determined also the cell number and proliferation by TC10 automated cell counter (Bio-Rad, Milan, Italy).

### 2.5. Morphological Evaluation of Cardiomyocytes by Confocal Microscopy

After 72 hr incubation with Tau, TMAO, and TMAO/Tau combination, the HG-H9c2 cells were fixed for 20 min with a 3% (*w*/*v*) paraformaldehyde (PFA) solution and permeabilized for 10 min with 0.1% (*w*/*v*) Triton X–100 in phosphate-buffered saline (PBS) at room temperature. To prevent nonspecific interactions of antibodies, cells were treated for 2 hr in 5% fetal bovine serum (FBS) in PBS; then, cells were incubated with a specific mouse monoclonal antibody raised against actin (1 : 500 Alexa Fluor®, BD Pharmingen™) for 24 hr at 37°C. The slides were mounted on microscope slides by Mowiol. The analyses were performed with a Zeiss LSM 510 microscope equipped with a plan-apochromat objective X 63 (NA 1.4) in oil immersion. Actin fluorescence was collected in a multitrack mode. Aggiungere dapi aggiungere nostra reference The nuclei were stained with 4′,6-diamidino-2-phenylindole (DAPI) [20].

### 2.6. Nitrite Levels

Nitrite was measured by the Griess reaction. Briefly, 50 *μ*L of medium was mixed with an equal volume of the Griess reagent (0.5% sulfanilamide, 2.5% H_3_PO_4_, and 0.05% naphthylethylene diamine in H_2_O) and incubated for 10 min at room temperature. Absorbance was assayed at 550 nm and compared with a standard curve obtained using sodium nitrite. [21]

### 2.7. Thiobarbituric Acid-Reactive Species (TBARS) Assay

Samples were incubated with 0.5 mL of 20% acetic acid, pH 3.5, and 0.5 mL of 0.78% aqueous solution of thiobarbituric acid. After heating at 95°C for 45 min, samples were centrifuged at 4000 rpm for 5 min. The TBARS were quantified by spectrophotometry at 532 nm. Results were expressed as TBARS *μ*M/g of proteins. Each data point is the average of triplicate measurements, with each experiment performed in triplicate.

### 2.8. AST and LDH Assay

NG-H9c2 and HG-H9c2 cardiomyocytes (1 × 10^5^ cells/well) after 24 and 72 hr incubation with Taurisolo®, TMAO, and TMAO/Tau combination were cultured in 6-well plates. The medium was collected for the measurement of the Aspartate transaminase (AST) and Lactate dehydrogenase (LDH) enzymes, including isoform 1 and 2 release. The enzyme activity was measured using an Abbott Aeroset fully automatic biochemical analyzer (Abbott Laboratories, USA). The levels of enzymes were assayed according to the instructions provided with the corresponding enzymatic kits.

### 2.9. Western Blots

We followed the methods of Vanacore et al. 2018 [22] for evaluation the protein expression by Western blot. Briefly, the cells were cultured at different condition for 72 hr, and then, cell pellets were lysed with 1 mL of lysis buffer (1% Triton, 0.5% sodium deoxycholate, 0.1 M NaCl, 1 mM Ethylenediamine tetra-acetic acid (EDTA), pH 7.5, 10 mM Na_2_HPO_4_, pH 7.4, 10 mM Phenylmethyl sulfonyl fluoride, 25 mM benzamidine, 1 mM leupeptin, and 0.025 units/mL aprotinin). The lysates were centrifuged at 12,000 rpm for 10 min at 4°C. Equal amounts of protein extracts were separated by SDS-PAGE, electrotransferred to nitrocellulose, and reacted with the different antibodies (ERK, pERK, LC3II, and Mn-SOD). All Western blots were repeated for three times. GAPDH was used as internal control. To quantify the results, the relative amount of each protein was determined.

### 2.10. Mass Spectrometry

LIPID MAPS Lipidomics Gateway and Human Metabolome Database queries were used to assign putative identities to mass features using based on mass accuracy within ±1 Da. (http://www.lipidmaps.org/data/structure/index.html.)

## 3. Results and Discussion

The present study was designed to test the ability of Taurisolo® to counteract or reduce the high glucose and TMAO-induced cardiomyocytes damage. All experiments were performed utilizing H9c2 cardiomyoblast cells growth in DMEM with 5.5 mM glucose (NG-H9c2), 44 mM glucose (HG-H9c2); both NG and HG-H9c2 cells were treated with 50 *μ*M TMAO (TNG-H9c2 THG-H9c2). We have set these concentrations of glucose and TMAO, because they produced moderate H9c2 cardiomyocyte toxicity and could be the conditions suitable for mimic in vitro diet-induced obesity (DIO) model.

### 3.1. Cardioprotective Effects of Taurisolo® in H9c2 Cells under TMAO-/High Glucose-Induced Cytotoxicity

**Figure 1:**
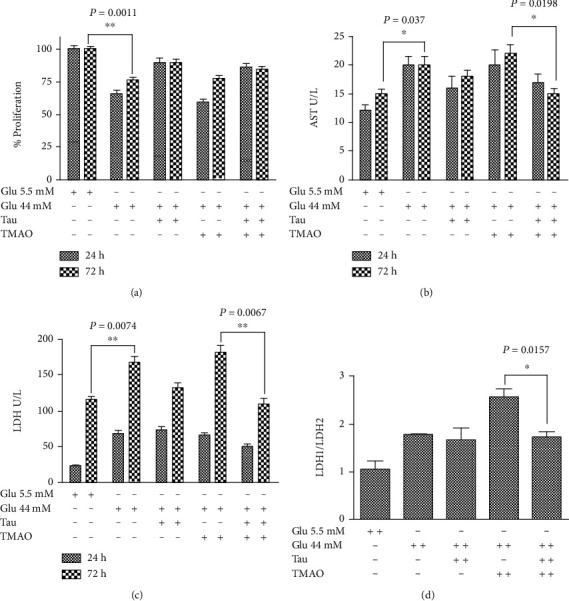
Taurisolo® (Tau) counteracts the H9c2 cell injury induced from TMAO in hyperglycemic condition. H9c2 cells were grown in standard (5.5 mM Glu NG-H9c2) and high glucose (44 mM Glu, HG-H9c2) condition. The HG-H9c2 cells were treated, for 72 h with Tau (0,5 *μ*g/*μ*L), TMAO(50 *μ*M), and TMAO-Tau combination. (a) The cell survival was performed with MTT assay. (b, c) AST and LDH as makers of cell injury were performed by the colorimetric assay. (d) LDH1/LDH2 isoenzymes ratio was evaluated at 72 h by the colorimetric assay. Each experiment was repeated at least three times. Results are expressed as mean ± SD.

To gain an insight about the mechanism through which Taurisolo® sustained the cardiomyocyte viability, we have damaged the H9c2 cardiomioblastic cell line with high glucose concentration (44 mM) and TMAO, a molecule linked to obesity and energy metabolism [23, 24] (Figure 1). H9c2 vitality was detected *via* MTT assay, while LDH and AST release assays were performed for cellular injury. High glucose treatment of H9c2 (44 mM) significantly reduced cell viability (~25%; *P* = 0.011) and increased both AST and LDH release (*P* = 0,037 and *P* = 0, 0074, respectively) compared with the NG-H9c2 growth in standard condition (5.5 mM glucose). In contrast, treatment with Taurisolo® was able to counteract significantly the decrease of vitality and the AST and LDH release in both HG-H9c2 and THG-H9c2 cells, while at the same concentration, Tau-treated NG-H9c2 did not produce the same effect.

**Figure 2:**
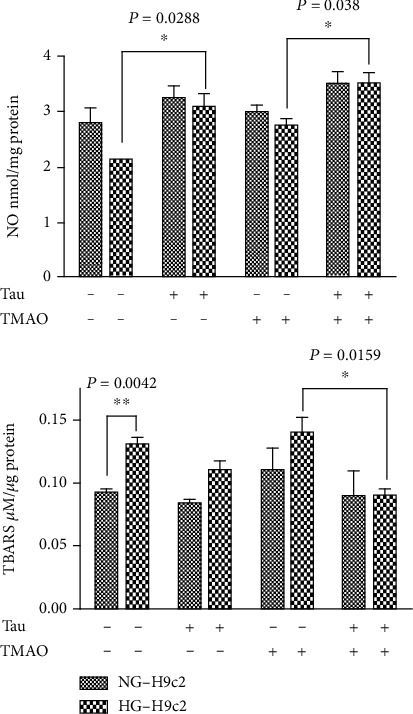
Taurisolo® increased the nitric oxide values in the medium of THG-treated H9c2 cells. The values of NO were evaluated by the Griess assay. H9c2 cells were grown in standard (5.5 mM NG-H9c2) and high glucose (44 mM Glu, HG-H9c2) condition. The NG and HG-H9c2 cells were treated, for 72 h with Tau (0.5 *μ*g/*μ*L), TMAO (50 *μ*M), and TMAO-Tau combination. Absorbance was assayed at 550 nm and compared with a standard curve obtained using sodium nitrite. TBARS were quantified by spectrophotometry at 532 nm. Results were expressed as TBARS M/g of serum proteins. Each data point is the average of triplicate measurements, with each experiment performed in triplicate. Results are expressed as mean ± SD.

**Figure 3:**
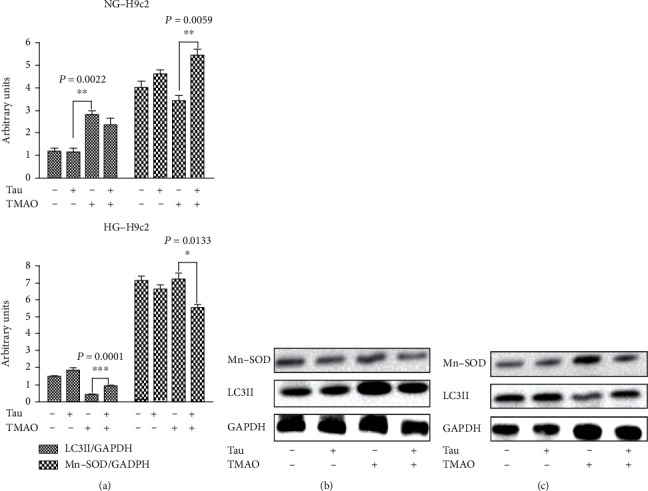
Taurisolo® (Tau) increase LC3II expression and counteract high levels of MnSOD. H9c2 cells were grown in standard (5.5 mM Glu NG-H9c2) and high glucose (44 mM Glu, HG9c2) condition. The NG and HG-H9c2 cells were treated, for 72 h with Tau (0.5 *μ*g/*μ*L), TMAO (50 *μ*M), and TMAO-Tau combination. (a) Bar graphs show densitometrically quantified LC3II, MnSOD with respect to house-keeping GAPDH in NG (5.5 mM glucose) and HG-H9c2 cells. Representative immunoblots of Tau treated TNG-H9c2 (b) and THG-H9c2 cells (c). Each data point is the average of triplicate measurements, with each experiment performed in triplicate. Results are expressed as mean ± SD.

In Figure 1(d), we reported the LDH1 : LDH2 isoenzyme ratio, observing that increased of about 1,8 and 2,5 fold in HG-H9c2 and THG-H9c2, respectively, compared to NG-H9c2 (LDH1 : LDH2ratio = 1). Interestingly, Taurisolo® treatment of THG-H9c2 counteracted the increase of LDH1/LDH2 ratio. To demonstrate that the effect of Taurisolo® on proliferation was related to the reduction of reactive oxygen species (ROS) production, we evaluated both lipid peroxidation by aldheide reactive to thiobarbituric acids (TBARS) assay and endogenous free nitric oxide (as NO_2_^−^) by the Griess assay (Figure 2). We observed a significant increase of TBARS production in THG-H9c2 cells compared to Taurisolo®-treated THG-H9c2 cells (*P* = 0.0159) and an increase of NO production in the medium of Taurisolo®-treated THG-H9C2 (*P* = 0.038) compared with untreated THG-H9c2 cells. Furthermore, Taurisolo® treatment counteracted the higher expression of Manganese Superoxide dismutase (Mn-SOD) in THG-H9c2 cells (Figure 3).

**Figure 4:**
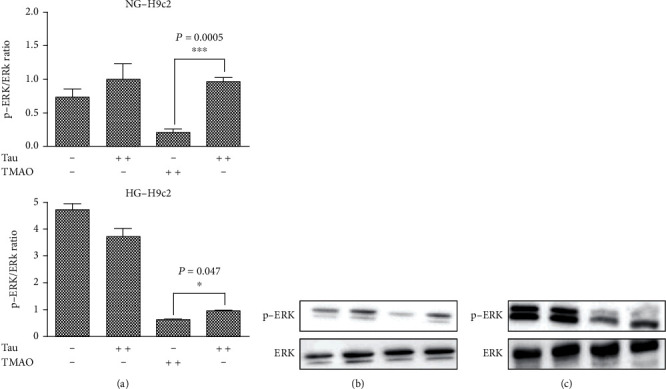
Taurisolo® modulated ERK activation. H9c2 cells were grown in standard (5.5 mM Glu NG-H9c2) and high glucose (44 mM Glu, HG9c2) condition. The NG and HG-H9c2 cells were treated, for 72 h with Tau (0.5 *μ*g/*μ*L), TMAO (50 *μ*M), and TMAO-Tau combination. (a) Bar graphs show densitometrically quantified ratio of p-ERK/ERK in H9c2 cells. (b) Representative immunoblots of NG-H9c2 cells treated with Tau, TMAO, and Tau-TMAO combination. (c) Representative immunoblots of HG-H9c2 cells treated with Tau, TMAO, and Tau-TMAO combination. Each data point is the average of triplicate measurements, with each experiment performed in triplicate. Results are expressed as mean ± SD.

Extracellular signal-regulated protein kinase (ERK) and its phosphorylated form (pERK) are important mediators of various cellular responses, such as proliferation, differentiation, and cell death. It is noting that chronic high glucose induced ERK phosphorylation and cell death. We detected ERK activation by evaluating the pERK/ERK ratio by Western blot analysis using phospho-specific antibodies. ERK activation (Figure 4(a)) was elevated in HG-H9c2 cells compared to NG-H9c2 cells (Figure 4), while it was significantly reduced after TMAO treatment in both NG and HG-H9c2 cells. Of note, treatment with Tau modulated ERK activation in prosurvival manner both HG and THG-H9c2 cells.

Collectively, these data suggested that the association of 72 h-hyperglycemia and 72 h-TMAO treatment of H9c2 cells led to both cardiac cell cytotoxicity and increase in OS, as mainly indicated by the increases of both lipid peroxidation and antioxidant enzyme levels. Taurisolo® treatment mitigated the increases in AST and LDH levels and increased H9c2 cell viability, suggesting its protective effect on cardiomyocytes during both hyperglycemia- and TMAO-caused damage. Furthermore, these results showed that Taurisolo® reduced THG-induced H9c2 injury by reducing OS. Decreased levels of TBARS are combined with increase of free NO production, suggesting a potential involvement of Taurisolo® in regulating the balance between NO and peroxynitrite. A protective effect of NO has also been observed in endothelial cells, cardiomyocytes [25], and inflammatory cells [26]. When MnSOD is overexpressed, more superoxide radicals are converted to H_2_O_2_, which acts as a cytotoxic agent, and therefore are removed from the physiological equilibrium, causing an increased production of membrane lipid peroxidation. To demonstrate that the cellular damage effect of high glucose and TMAO was related to increased oxidative stress, we assessed cell vitality after treatment for 24 h with 10 *μ*M N-acetyl cysteine (NAC) widely used as a pharmacological antioxidant and cytoprotective compound. In this experimental condition, we did not observe growth inhibition in both HG and THG-H9c2 cells (data not shown). In conclusion, the protective effect of Taurisolo® on cell viability and cell injury is closely linked to the antioxidant activity of its polyphenol composition. To demonstrate that the antiproliferative effect inducted in high glucose concentration was related to increased oxidative stress, we assessed cell vitality after treatment for 24 h with 10 *μ*M NAC. In this experimental condition, we did not observe growth inhibition in both HG and THG-H9c2 cells (data not shown) compared to NG-H9c2 cells. In conclusion, the protective effect of Taurisolo® on cell viability and cell injury is closely linked to the antioxidant activity of its polyphenol composition. The antioxidant potential of Taurisolo® results in decreased both expression of MnSOD (Figures 3(a)–3(c)) and lipid peroxidation levels. Conversely, the increased release of NO leads to an ERK activation and a consequently prosurvival effect against TMAO damage induced in the H9c2 cells.

.

### 3.2. Taurisolo® Increases Autophagic Process and Remodeling *α*-Actin Distribution under High Glucose- and TMAO-Induced H9c2 Injury

**Figure 5:**
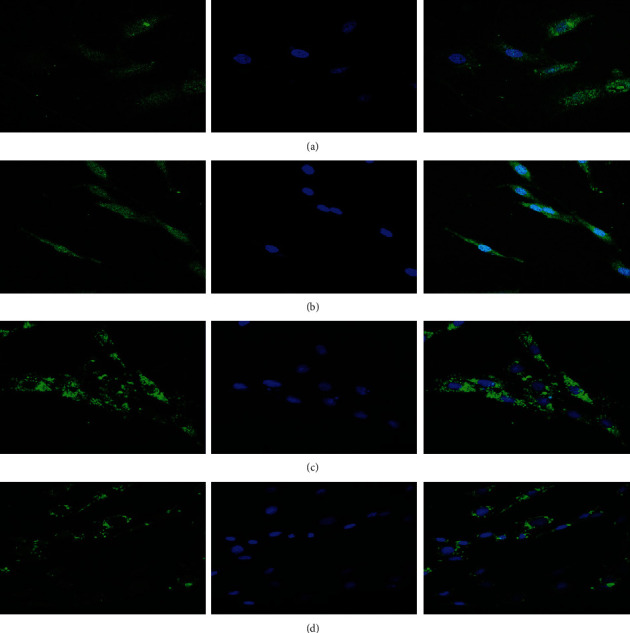
Representative microphotographs of HG-H9c2 cells. Cardiomyocyte was identified with *α*-actin antibody (green signal), and the nucleus was identified by DAPI (blue signal). (a) H9c2 cell growth with 44 mM glucose (HG-H9c2). (b) HG-H9c2 was treated for 72 h with Tau (0.5 *μ*g/*μ*L). (c) HG-H9c2 cell was treated for 72 h with TMAO (50 *μ*M). (d) HG-H9c2 cell was treated for 72 h with TMAO/Tau combination.

As autophagy is important for the maintenance of mitochondrial homeostasis, it was hypothesized that Taurisolo® may preserve the H9c2 from injury induced by TMAO and hyperglycemia *via* autophagy activation. To prove this hypothesis, we evaluated the autophagy marker protein microtubule-associated protein LC3II expression by western blot analysis. TMAO treatment of HG-H9c2 induced a downregulation of autophagy, as evidenced by a decrease in LC3II, compared with the HG-H9c2 cells. 72 h-treatment with TMAO/Taurisolo® combination of HG-H9c2 cells was able to reverse the decrease of LC3II expression in THG-H9c2 cells (Figures 3(a)–3(c)). Morphological changes induced by both HG and TMAO in H9c2 cells were detected by *α*-actin immunofluorescence by confocal microscopy; the representative results were shown in Figure 5. The *α*-actin protein (green signal) in Taurisolo®-treated HG-H9c2 cells was uniformly distributed in the cytoplasm, and cells presented a typical elongated form with respect to untreated HG-H9c2 cells where the shape appeared enlarged and rounded; in THG-H9c2 cells, *α*-actin accumulated, forming evident punctuated signals. Meanwhile, Taurisolo® treatment reduced actin aggregation in THG-H9c2 cells. These results demonstrate that exposure to Taurisolo® in both HG and THG-H9c2 cells induced *α*-actin spatial organization and a functional cell morphological conformation. The purpose of autophagy is to ensure quality control of organelles and proteins, as well as protection of intracellular homeostasis in stress and nutrient efficiency [27–32]. Autophagy is involved in the maintenance of organelle integrity, protein quality [33], and modulated and to participate in the pathogenesis of human diseases, such as DM, neurodegenerative diseases, aging, and vascular disease [7, 34, 35]. It has been reported that antioxidant molecules such as resveratrol by increasing autophagic flux ameliorates diabetic cardiomyopathy [36]. In THG-H9c2 damage cell model cells, we demonstrated that Taurisolo®, restoring the autophagic process, induces a reduction of the actin aggregation, restoring a normal cell morphology.

### 3.3. Taurisolo® Induces the *Ex Novo* Synthesis of Sphingomyelin, Ceramides, and Their Metabolites

**Figure 6:**
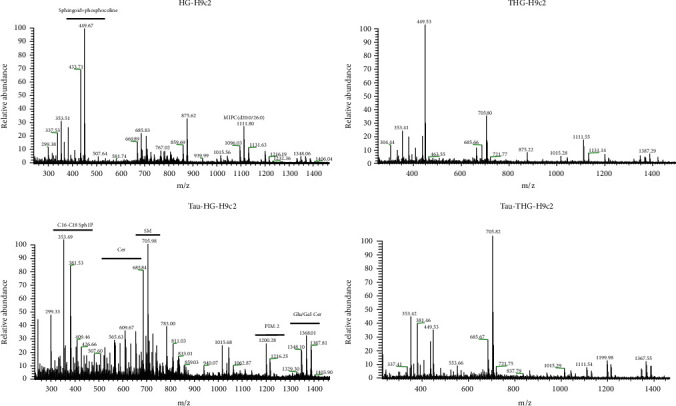
Positive mass spectra of lipid extracted from media of H9c2 cells. Positive ion electrospray mass spectra of lipid molecular species in lipid extracts from medium of the HG-H9c2 (44 mM glucose) and THG-H9c2 (44 mM glucose + 50 *μ*M TMAO) before and after Tau treatment (Tau-HG-H9c2; Tau-THG-H9c2). Aliquots of chloroform extracts were analyzed directly by electrospray as described in the Materials and Methods section. Selected peaks are indicated by their m/z values. For detailed peak assignments, see Table 1 (Supplementary Materials).

SLs are molecules implicated in cell survival and autophagy. Bioactive lysolipids, including Cer and sphingosine-1-phosphate, may act as both extracellular and intracellular mediators. We used tandem mass spectrometry to investigate which sphingolipids were secreted in the cell medium in Taurisolo®-treated HG- and THG-H9c2 cells. In Figure 6, the positive ion mass spectrometry profile of lipid extract in the medium of the HG-H9c2, THG-H9c2, and both Taurisolo®-treated HG- and THG-H9c2 cells was reported. In both HG- and THG-H9c2 spectra, we identified the following peaks: d18: 1 sphingoid base and phosphocholine headgroup (449 m/z) [37] (Monounsaturated 18-carbon dihydroxylated sphingoid base linked to one chain of palmitic acid denominated 1-O-tricosanoyl-Cer (d18: 1/16: 0) (875 m/z) and N-(hexacosanoyl)-eicosasphinganine-1-O-[D-mannopyranosyl-*α*1-2-myo-inositol-1-phosphate] MIPC (d20 : 0/26 : 0) (1111 m/z). We observed in the lipid fingerprint profile of Tau-treated HG- and THG-H9c2 cells that the metabolite peaks were centered between 200 and 1500 m/z as sphingosine, C16-C18 sphinganine-1-phosphate, PE-Cer (d16 : 2/20 : 1), SM (d18 : 0/16 : 0), PI-Cer (d18 : 0/16 : 0), 1-O-stearoyl-Cer (d18 : 1/18 : 0)/Or 1-O-eicosanoyl-Cer (d18 : 1/16 : 0), MIPC (18 : /20), PIM2 (16 : 0/18 : 1), and Glu/Gal-ceramide. Cer plays a central role in sphingolipid metabolism. Cer consists of sphingoid long-chain base linked to an acyl chain *via* an amide bond and synthesized *de novo* in the endoplasmatic reticulum (ER); it can be modified into Golgi in sphingomyelin (SM), sphingosine, and glycosphingolipids (e.g., galactosylceramide) and are transported to the plasma membrane (PM). Cer can then be metabolized into ceramide-1-phosphate (C1P) and sphingosine-1-phosphate (S1P) or be resynthesized back into SM. Sphingosine was associated with growth arrest [38] (whereas SP1 promoted cell proliferation and prevents programmed cell death [39]). In H9c2 cells, higher glucose concentration may block the Cer production and then promote transformation in SM and Sph1P, whereas Taurisolo® treatment of HG and THG-H9c2 cells induced a reprogramming lipid metabolism and increased Cer, SM, and Sph1P productions that may protect the cardiomyocyte from glucose cytotoxicity. Further *in vivo* researches may serve to license Taurisolo® as a useful nutraceutical approach in the prevention of heart damage in obese subjects.

## 4. Conclusion

It has been previously reported that polyphenols enhance glucose uptake, mainly in the muscular tissue, *via* increasing expression and/or translocation of GLUT4 transporters [40–42]. Moreover, studies demonstrated that polyphenols Resveratrol and Quercetin exert antioxidant and anti-inflammatory properties [43–46] and can ameliorate the diabetic complications [47]. However, the beneficial effects of polyphenolic compounds in functional foods and nutraceuticals are affected by several factors compromising their bioavailability. Notably, several strategies have been developed by the pharmaceutical and nutraceutical industries in order to counteract this crucial concern, including microencapsulation in various coating materials. Microencapsulation, indeed, has been demonstrated to (i) protect polyphenols against degrading factors met during the transit in the gastrointestinal tract, including pH variations and digestive enzymes (increasing, thus, their bioaccessibility) and (ii) favor their absorption across the intestinal barrier (increasing their bioavailability) [48]. In this sense, Taurisolo® is a nutraceutical formulation based on grape polyphenols microencapsulated in maltodextrins. In our previous studies [16], we demonstrated high bioavailability of Taurisolo® polyphenols, leading to assume that also after oral administration, these bioactive compounds may reach high tissue concentrations.

Annunziata and colleagues [16, 17] have demonstrated the ability of the Taurisolo® to reduce circulating levels of TMAO, a molecule related to metabolic syndrome and cardiovascular risk [49]. Moreover, the TMAO levels are inversely associated with the adherence to the Mediterranean diet [50]. The present study was designed to test the ability of Taurisolo® to counteract or reduce the cardiomyocytes damage induced by high glucose concentration and TMAO. The hyperglycemia in ventricular cardiomyoblasts H9c2 cell induces cytotoxicity, OS, and cellular hypertrophy, whereas TMAO reduces autophagy and causes actin accumulation. Taurisolo® exerted a protective effect on both hyperglycemia and TMAO-induced H9c2 cell injury by (1) modulation of OS through its direct scavenger action, (2) induction of morphological changes by *α*-actin redistribution, and (3) activation of the autophagic process by means of the *ex novo* synthesis of Cers and their metabolites [51]. Here, we demonstrated that Taurisolo® improves *in vitro* HG- and TMAO-induced H9c2 dysfunction, suggesting that Taurisolo®, combined with energy restriction, may represent a useful nutraceutical approach for the prevention of cardiomyopathy in obese subjects.

## Data Availability

The data used to support the findings of this study are available from the corresponding author upon request.

## Abbreviations

Sph: Sphingosine
Sph1P: Sphingosine 1-phosphate
Cer: Ceramide
Cer1P: Ceramide 1-phosphate
GalCer: Galactosylceramide
GlcCer: Glucosylceramide
LacCer: Lactosylceramide
GM, GD, GT: Gangliosides
SM: Sphingomyelin
LC-MS/MS: Liquid chromatography tandem mass spectrometry
Q: Quadrupole
QQQ: Triple quadrupole
QTrap: Quadrupole linearion trap
PI-P: Phospho-(1′-myo-inositol)
LC3: Microtubule-associated protein 1 light chain 3.

## Supplementary Materials

Supplementary Materials Assignments of the *m*/*z* ratios detected in the positive ion mass spectra of the lipid extracts in the growth medium of HG-H9c2 (44 mM glucose) and THG-H9c2 (44 mMglucose+TMAO 50 *μ*M) cells before and after treatment with Tau 0.5 *μ*g/*μ*L.
