# Supplementary material for: Cardioprotective Effects of Taurisolo® in Cardiomyoblast H9c2 Cells under High-Glucose and Trimethylamine N-Oxide Treatment via De Novo Sphingolipid Synthesis
Source: Oxid Med Cell Longev. 2020 Nov 12;2020:2961406. doi: 10.1155/2020/2961406 (PMC7683148; doi:10.1155/2020/2961406)
Supplement: Supplementary Materials — Assignments of the m/z ratios detected in the positive ion mass spectra of the lipid extracts in the growth medium of HG-H9c2 (44 mM glucose) and THG-H9c2 (44 mMglucose+TMAO 50 μM) cells before and after treatment with Tau 0.5 μg/μL. [file 2961406.f1.pdf]

Supplementary Material

In the Table1 we reported the *m/z* and the identity of the most representative ion positive peaks of lipid extracted of the medium of H9c2.

Table 1: Assignments of the *m/z* ratios detected in the positive ion mass spectra of the lipid extracts in the growth medium of HG-H9c2 (44mM glucose) and THG-H9c2 (44 mMglucose+TMAO 50 µM) cells before and after treatment with Tau 0.5µg/µL .

m/z	**Identity**
353.1±1	C-16 Sph1P
381.3±1	C-18 Sph1P
449±1	d18: 1 sphingoid base and phosphocholine headgroup
709±1	Sphingomyelin (SM)
685±1	Phosphoethanolamine-Cer (PE-Cer)
783±1	1-Phospho-(1'-myo-inositol)-Cer(d18:0/16:0)
1111±1	MIPC(N-(hexacosanoyl)-eicosasphinganine-1-O-[D-mannopyranosyl-alpha1-2-myo-inositol-1-phosphate]
1200±1	PIM2 (2'-O-(alpha-D-Manp)-6'-O-(alpha-D-Manp)-(1-(9Z,12Z-nonadecadienoyl)-2-(9Z-octadecenoyl)-sn-glycero-3-phospho-1'-myo-inositol)
1368±1	Glu/Gal-Ceramide
